# Deciphering transcription factor binding patterns from genome-wide high density ChIP-chip tiling array data

**DOI:** 10.1186/1753-6561-5-S2-S8

**Published:** 2011-05-28

**Authors:** Juntao Li, Lei Zhu, Majid Eshaghi, Jianhua Liu, Krishna Murthy R Karuturi

**Affiliations:** 1 Computational & Systems Biology, Genome Institute of Singapore, 60 Biopolis Street, (S)138672, Singapore; 2 Systems Biology, Genome Institute of Singapore, 60 Biopolis Street, (S)138672, Singapore

## Abstract

**Background:**

The binding events of DNA-interacting proteins and their patterns can be extensively characterized by high density ChIP-chip tiling array data. The characteristics of the binding events could be different for different transcription factors. They may even vary for a given transcription factor among different interaction loci. The knowledge of binding sites and binding occupancy patterns are all very useful to understand the DNA-protein interaction and its role in the transcriptional regulation of genes.

**Results:**

In the view of the complexity of the DNA-protein interaction and the opportunity offered by high density tiled ChIP-chip data, we present a statistical procedure which focuses on identifying the interaction signal regions instead of signal peaks using moving window binomial testing method and deconvolving the patterns of interaction using peakedness and skewness scores. We analyzed ChIP-chip data of 4 different DNA interacting proteins including transcription factors and RNA polymerase in fission yeast using our procedure. Our analysis revealed the variation of binding patterns within and across different DNA interacting proteins. We present their utility in understanding transcriptional regulation from ChIP-chip data.

**Conclusions:**

Our method can successfully detect the signal regions and characterize the binding patterns in ChIP-chip data which help appropriate analysis of the ChIP-chip data.

## Background

With the microarray technology rapidly advanced, tiling arrays have quickly become one of the most powerful tools in genome-wide investigations. High density tiling arrays [1] can be used to address many biological problems such as transcriptome mapping, protein-DNA interaction mapping (ChIP-chip) and array CGH among others [2]. ChIP-chip [3], the focus of the paper, is a technique that combines chromatin immunoprecipitation (ChIP) with microarray technology (chip). It allows efficient, scalable and comprehensive identification of binding sites and profiles of DNA-binding proteins [4]. High density ChIP-chip tiling arrays not only help us map the binding sites of a protein in the genome, but also allow us to better understand the binding events of the protein by clearly displaying the binding occupancy profiles. Several methods have been proposed to analyze the ChIP-chip data; for example, Joint Binding De-convolution (JBD) [5] uses a probabilistic graphical model to improve spatial resolution of identification of the transcription factor binding sites. However it requires the DNA fragment length distribution which may not always be available. Its usefulness may be limited for high density tiling array since the resolution is considerably high. MPeak [6,7] fits a mixture of triangular basis to model the binding or interaction data. It ignores the complexity of binding event and only roughly characterizes the basic patterns for single and direct binding events. The more complex binding patterns from high density ChIP-chip may not be well explained using mixture of a triangular basis. Model-based Analysis of Tiling-arrays (MAT) [8] reliably detects the signal enriched regions on Affymetrix tiling arrays by considering the probe sequence and copy number of all probes on a single tiling array. The aim of those methods is to locate the binding sites only and not to characterize different binding profiles present in different genomic regions.

In the view of the complexity of the DNA-protein interaction, the opportunity offered by high-density tiled ChIP-chip data and the lack of methods to exploit wealth of information in the ChIP-chip data, we present a new statistical procedure to analyze high density ChIP-chip tiling array data to characterize protein-DNA interaction in terms of binding sites as well as the binding profiles. First, we identify the enriched ChIP signal regions or protein binding occupancies using moving window binomial analysis and split the signal regions with multiple peaks into individual peak regions. Second, the signal regions are classified into two categories using peakedness test and analyzed separately. Third, the peak regions are analyzed to get the peak positions signifying the most probable binding sites, and using skewness assessment to improve the peak assignment to genes. The flat binding occupancies are processed to summarize their overall strength, their peak location is irrelevant as flat occupancies signify non-specific binding to any particular locus within its range.

In this article, we applied our procedure to analyze the high density ChlP-chip data of fission yeast (*Schizosaccharomyces pombe*) obtained from custom designed NimbleGen genome tiling arrays of ~380*k* probes. We studied two stress-related transcriptional factors (TFs) Pcr1 and Atf1 with H_2_O_2_ treatment [9], and one general transcription factor Tbp1 (TATA box binding protein). We also included the RNA polymerase II large subunit, Rpb1 (with and without H_2_O_2_ treatment), which is used to indicate transcriptionally active genes of *S.pombe* (Table1). We found that DNA-binding proteins show distinct patterns in the proportions of peak and flat binding profiles. Tbp1 and stress-related TFs show more peak binding patterns indicating their location specific binding, and Rpb1 present a large fraction of flat signal regions within protein-coding regions indicating its non-specific binding along gene body.

**Table 1:** The list of TFs studied.

Protein	Description	Condition	Repeats
Atf1	transcription factor Atf1. Transcription factor required for sexual development and entry into stationary phase.	H_2_O_2_	1
Pcr1	Transcription factor Pcr1. Involved in regulation of gene expression for sexual development.	H_2_O_2_	1
Tbp1	TATA-binding protein (TBP). General transcription factor that functions at the core of the DNA-binding multiprotein factor TFIID.	Normal	2
Rpb1	RNA polymerase II large subunit Rpb1.	Normal	2
Rpb1	RNA polymerase II large subunit Rpb1.	H_2_O_2_	2

## Methods

**Figure 1:**
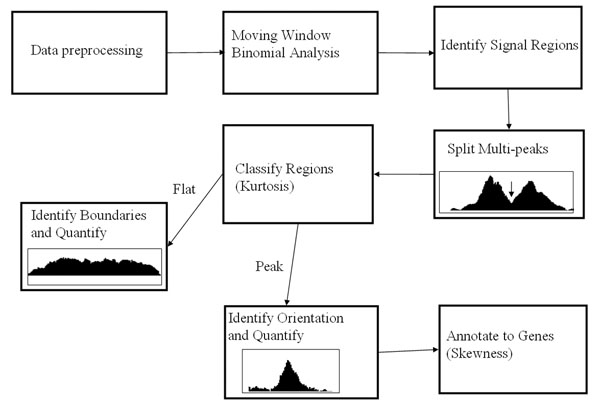
The proposed statistical procedure for ChIP-chip data analysis.

The tiling array has very high resolution and probes cover the whole genome, and ChIP procedure selects only protein binding sites which are a small part of the genome. Therefore, only a very small proportion of probes in tiling array has the binding signal and majority of probes’ signals will be close to the background. Hence we median centered the log transformed data for further analysis. The proposed statistical procedure is illustrated in Figure 1.

### Moving window binomial analysis

For any probe in location *i*, let *x_i_* (*i* = 1,…, *n*) denote the median centered log signal. We define the base threshold *c*MAD, *c* fold MAD (median absolute deviation) of all *x_i_*’s in the array, for *x_i_* (*i* = 1,…, *n*), then

is the probability that the signal of a probe passes the base threshold. Where #{*x_i_|x_i_* ≥ *c*MAD} is the total number of probes whose signals pass the base threshold.

Then *p_w_*(*x_i_*) is defined as the probability that *x_i_* is classified as signal by considering its neighborhood region  as the signal region, and can be computed by binomial testing as in the following equation,

where *L* is the number of probes above the base threshold in the region  and *w* is the predefined half window size.

We define a region  as a signal region if

where *α* is the *p*-value cutoff for the binomial test.

After having identified signal regions, the task is to process them to identify binding sites and their profiles. The pre-requisite is to smooth the signals to reduce noise by smoothing the signal regions and their neighborhoods. Two different smoothing methods, multiple round moving average and median smoothing, are employed to reduce the noise in the data. Multiple round moving average smoothing method will retain the signal shape and the peak signal (local maximum) loci. This method has already been used in the ChIP-chip peak finder [10]. We use moving average method to split multi-peak signal regions, identify the peak loci and compute kurtosis and skewness scores of signal regions. The drawback of moving average is that it may destroy the boundaries of the signal regions, therefore we apply moving median smoothing method to characterize signal regions.

#### Splitting multi-peak signal regions

Each signal region from moving window binomial analysis may contain multiple peaks (local maxima), indicating multiple binding sites, and this will make the binding patterns more complex. Therefore, the regions with multiple peaks are split at the troughs of their profiles. For doing this, we first assign each probe *x_i_* of smoothed profile one of the *γ_x_i__* = {+, –, 0} to indicate whether the binding signals significantly increased, decreased or not significantly changed from the immediately neighboring probes,

where *d* = MAD(*δ_i_*), *δ_i_* = |*x_i_* – *x_i_*_+1_| for *i* = 1, 2,…, *n* – 1. After removing all probes with ”0”, the region is split at the transition of signs from “-” to “+”. After splitting, the signal regions of less than 4 probes are removed since those short signal regions generally have week signals and cannot be characterized by peakedness and skewness assessment.

#### Peak position identification

Peak position of a signal region with one significant peak is determined after the moving average smoothing of the profile. The position of probe with the maximal value in this region is defined as the position of the peak, the binding site.

### Peakedness and skewness assessment of signal regions

If we consider the smoothed signal region  as a probability mass function, it can be assessed for peakedness using its kurtosis score (*K*) using the formula

where  and . The region is designated as having flat-shape when its *K <* 2. Thus, peaked regions are separated from flat regions.

Similarly, for single peak regions, we used skewness score to test whether the peaks are left-skewed or right-skewed which indicates the binding orientation. This is very useful for transcription factor binding assignment to a single gene if there is a binding region in a bidirectional intergenic region (intergenic regions from divergent pair of genes). The skewness score (*G*) is calculated using the formula

where the *u* and *p_j_* have same definition as in kurtosis definition.

## Results

We used data from customized NimbleGen Tiling array designed for *S. pombe* (fission yeast) which has ~380*k* 50*mer* probes. They cover both strands of entire *S. pombe* genome based on the genome sequence from Wellcome Trust Sanger Institute (ftp://ftp.sanger.ac.uk/pub/yeast/pombe/). In each strand, there is a 16*bp* interval between two consecutive probes. The probes on the reverse strand are placed so that they cover the gaps between consecutive probes of the pairing forward strand. Therefore, the probes have 17*bp* overlap with each other. The probes with multiple hits in the genome will have the same level of signals in multiple loci and they cannot distinguish which locus contributed to the signal. Therefore, the probes with more than 4 hits were removed from the analysis, only ~2% of probes have been removed.

### Transcription factor binding regions and binding patterns

We analyzed ChIP-chip experiments of three DNA-binding proteins: two stress-related transcription factors Atf1 and Pcr1, one general transcription factor Tbp1 together with the RNA polymerase II large subunit Rpb1 whose occupancy indicates transcriptionally active genes. Tbp1 is a core subunit of the eukaryotic transcription factor TFIID which binds specifically to the TATA box. It contributes to load and release of RNA polymerase II at the transcription start sites (TSS). Furthermore, Tbp1 is also a necessary component of RNA polymerase I and RNA polymerase III. Therefore, Tbp1 is a good choice for binding pattern study. There are two replicates of ChIP-chip experiment for Tbp1 and Rpb1, and one replicate for Atf1 and Pcr1 with H2O2 treatment [9]. The data of Tbp1 and Rpb1 will be available in the upcoming publications. The signal regions for each array were identified with the stringent criteria of *c*MAD= 2MAD at *p*-value less than 0.001 (*α* = 0.001). The number of signal regions is ~ 1600 (~ 1300 before multi-peak splitting) for each replicate of Tbp1, ~ 1000 (~800 before multi-peak splitting) for Atf1/Pcr1, and for Rpb1 it is ~ 800 (~ 500 before multi-peak splitting). We applied Dice coefficient to measure the similarity of signal regions between two repeats.

where |*A*| and |*B*| is the total length of all signal regions of the first and second repeats, |*A* ∩ *B*| is the length of their overlapping regions. The coefficient for Tbp1 is 0.921 which indicates that our results of Tbp1 signal regions are highly reproducible. The coefficient for Rpb1 is 0.743 which is still considerably high.

**Figure 2:**
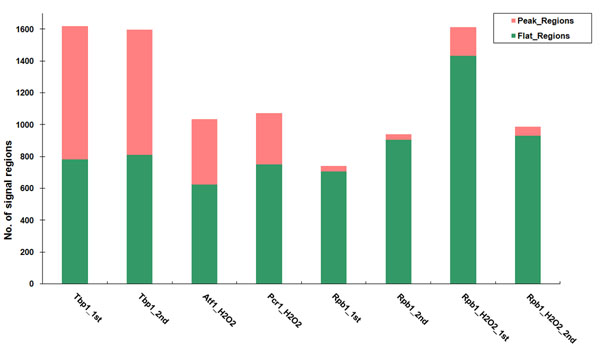
The signal regions summary for ChIP-chip datasets.

The summary of the kurtosis score is shown in Figure 2. More than half of the transcriptional factors (including Tbp1, Atf1 and Pcr1) signal regions are sharp peaks and Rpb1 (with and without H_2_O_2_ treatment) signal regions are mostly flat. It is consistent with our knowledge about the characteristics of those DNA-binding proteins. Since the purpose of performing a ChIP-chip experiment is to transform transcription factor binding sites into IP-enriched DNA, the specificity of protein-DNA binding finally determines the peaks of IP-enrichment. Therefore, due to the specific binding at TSS position, Tbp1 binding events were observed to result in many sharp peaked signal regions. Similarly, Atf1/Pcr1 also display many sharp peaked signal regions which is consistent with the belief that they are to be active at the promoter regions of their target genes and show specific binding sites. However, Rpb1 mostly presents flat occupancies in coding regions because of the function of Rpb1 which controls transcription elongation and synthesizes messenger RNAs.

To conduct more reliable analysis, we used the common Tbp1 bindings between replicated experiments. The common binding was defined as a binding region from the 1st experiment having the overlapping region from the 2nd experiment and the binding peaks of these two overlapping regions are both located inside of the intersection region. To measure the level of transcription activity of each gene, we detected the median level of Rpb1 occupancies within a coding region and used the average of the replicated experiments as a measure of the transcription level.

**Figure 3:**
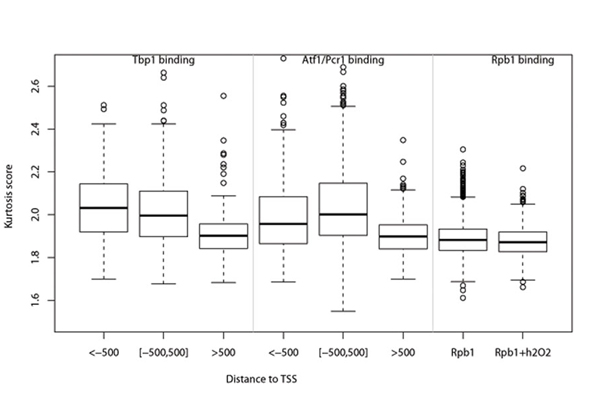
The kurtosis score distribution for TF (Atf1, Pcr1 and Tbp1) occupancies in the three genomic regions: <-500 (the upstream intergenic regions beyond 500bp from TSS),[–500, 500] (regions within 500bp of TSS) and >500 (the downstream intragenic regions beyond 500bp from TSS). And kurtosis score distribution for Rpb1 (with and without H_2_O_2_ treatment put together) binding.

We investigated the kurtosis score distribution for all three transcription factors (Atf1, Pcr1 and Tbp1) in three different genomic regions based on *pombe* genome TSS definition [11]: regions within 500bp of TSS ([–500, 500]), the upstream intergenic regions beyond 500bp from TSS (<-500) and the downstream intragenic regions beyond 500bp from TSS (>500). As shown in Figure 3, higher kurtosis scores are observed in the regions of [–500, 500] and <-500 where are mostly promoter regions. it implies that transcriptional factors favor to bind these two regions. On the other hand, the TF signal regions falling into >500bp downstream of TSS (mostly are coding regions) have low kurtosis compared with the promoter regions (*p*-value=1.620e-10 in Tbp and *p*-value<2.2e-16 in Atf1/Pcr1), and the binding signals in these regions are generally weak and unstable. The low kurtosis signals in coding regions is probably due to the interaction between TFs and RNA polymerase and the whole complex were involved in transcription elongation. The Rpb1 binding regions mostly fall into coding regions and present the flat patterns with low kurtosis.

### TF binding affinity positively correlates with the gene transcription level

**Figure 4:**
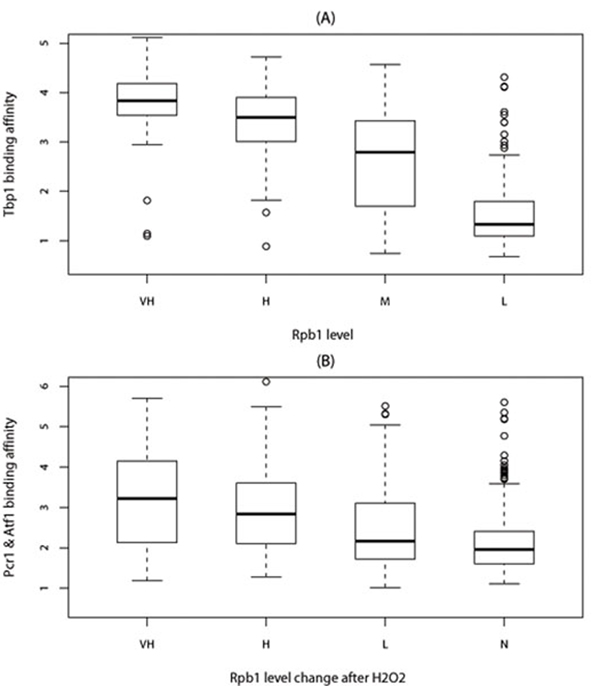
(A)Tbp1 binding affinity for VH(very high, Rpb1≥2), H(high, 1.5≤Rpb1<2), M(Median, 1≤Rpb1<1.5) and L(Low, 0.5 ≤Rpb1< 1) Rpb1 level groups. (B)Atf1 and Pcr1 binding affinity for VH(very high, ∆Rpb1≥2), H(high, 1≤∆Rpb1<2), L(Low, 0≤∆Rpb1<1), N(No, ∆Rpb1< 0) Rpb1 level change after H_2_O_2_ treatment groups. Where ∆Rpb1 = Rpb1_H_2___O_2__ - Rpb1 (Rpb1_H_2___O_2__ and Rpb1 are Rpb1 level with and without H_2_O_2_ treatment).

After having identified all peaked signal regions from ChIP-chip data, the next step is to map those regions to genes. To our knowledge, there is no perfect method to accurately map binding sites to genes. Therefore, we limited our investigation to the peaks only from the unidirectional intergenic (IGU) regions which are easy to assign, i.e. assigned to the downstream genes, to reduce the risk of assignment errors. Furthermore, we filtered out peaks not within the upstream 1*kb* of any gene as it may be out of the promoter regions for *S.pombe*. The upstream peaks of any RNA genes have also been removed since Tbp1 is also associated with RNA polymerase I and RNA polymerase III. We chose the highest peak if one promoter region have multiple peaks. There are 379, 165 and 172 peaks of Tbp1, Atf1 and Pcr1 respectively in IGU regions (IGU peaks) after filtering. Then we investigated the relationship between TFs binding affinity and transcription levels. From Figure 4(A), we observed positive correlation between Tbp1 binding affinity and transcription levels of the protein-coding genes (correlation is 0.55 and *p*-value<2.2e-16). It implies that the highly transcribed genes tend to be initiated by high Tbp1 binding affinity at their promoters. Similar to Tbp1, we also observed positive correlation between Atf1/Pcr1 binding affinity and the Rpb1 level change after H2O2 treatment (Figure 4(B), correlation is 0.30 and *p*-value=1.110e-15).

### Skewness of Tbp1 binding regions helps identifying Tbp1 regulated genes

**Figure 5:**
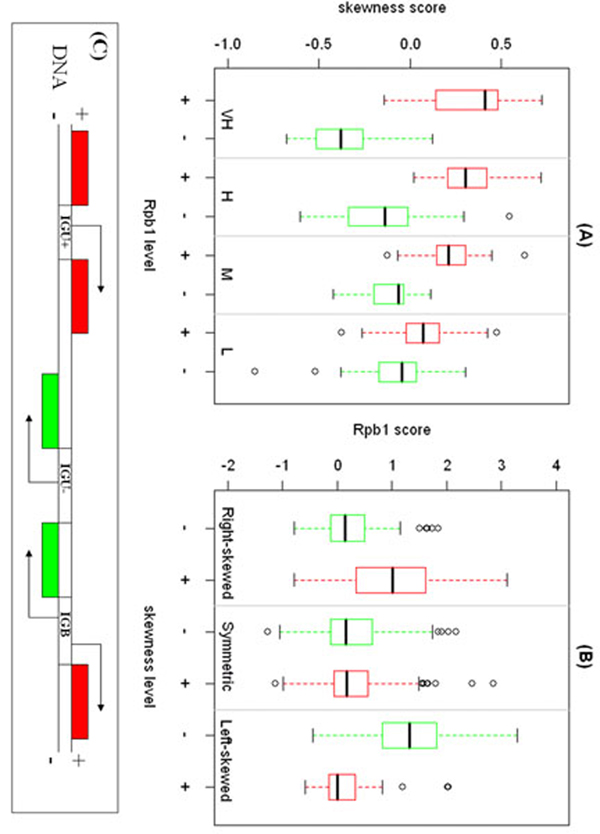
(A)The skewness score of Tbp1 binding in IGU (unidirectional intergenic) regions for VH(very high, Rpb1≥2), H(high, 1.5≤Rpb1<2), M(Median, 1≤Rpb1<1.5) and L(Low, 0 ≤Rpb1< 1) Rpb1 level groups. Red boxes are the IGU+ regions and green boxes are IGU- regions. (B)The Rpb1 score for Right-skewed, Symmetric and Left-skewed groups in IGB (bidirectional intergenic) regions. Red boxes are genes in forward strand and green boxes are genes in reverse strand. (C)The illustration of the IGU between forward strand genes (IGU+), reverse strand genes (IGU-) and IGB regions. Red boxes are genes in forward strand and green boxes are genes in reverse strand.

The skewness scores of Tbp1 binding regions also positively correlate with Rpb1 occupancy levels. We investigated the skewness for 379 IGU Tbp1 peaks in unidirectional intergenic regions. Interestingly, the patterns of Tbp1 binding skewed towards the direction of the immediate downstream gene which has transcription event. In other words, Tbp1 signal region displays an extended tail into the ORF of its target gene. As shown in Figure 5(A), transcribed genes on the forward strand tend to display positive skewness for Tbp1 binding occupancies in their promoters regions, and the transcribed genes on the reverse strand preferentially show negative skewness. Another interesting observation is that the absolute skewness declines with the decreasing Rpb1 level of the downstream genes indicating that Tbp1 binding pattern is enough to predict whether the downstream genes are transcribed in that condition. Our explanation is that Tbp1 may have persistent interaction with RNA polymerase in transcribing regions during the transition between transcription initiation and elongation, and the Tbp1 occupancy would correlate with the transcription rate of the downstream genes.

To further test our observations and demonstrate the utility of the skewness of binding regions, we checked the correlation between skewness of Tbp1 binding in the bi-directional promoters and Rpb1 levels of the flanking genes. The assignment of binding sites in the bidirectional promoters is always a problem: some studies assign them to both genes while the others assign them to the nearest gene. Here, we found skewness score may help us to get better assignment i.e. to identify the genes activated by Tbp1. In order to be conservative, we removed bidirectional intergenic regions with one of the flanking genes is an RNA gene and also discarded those gene pairs having more than one Tbp1 binding peaks. Finally there were 387 gene pairs used in the analysis. As shown in Figure 5(B), when the skewness scores are significantly positive (greater than 0.15) then the transcription level of the genes on the forward strands is clearly higher compared to the corresponding paired gene on the reverse strand, vice versa.

**Figure 6:**
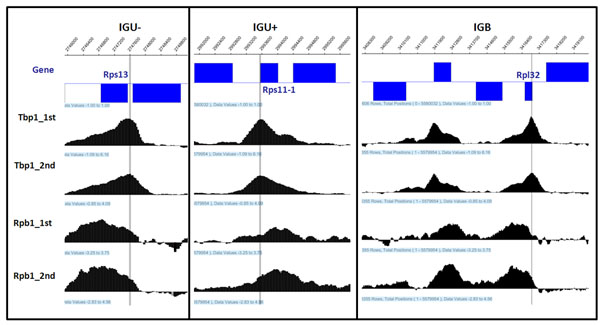
Three Examples of Tbp1 binding patterns and Rpb1 occupancies with two repeats in IGU+, IGU- and IGB regions. The blue boxes in first track indicate the gene ORF regions, and the vertical lines indicate the peak loci for Tbp1 binding.

When the binding pattern seems symmetric (between -0.15 and 0.15), there are no significant differences for transcription level between genes on the forward strand and the reverse strand. In addition, as shown in Figure 5, the presence of symmetric pattern is associated with the low Rpb1 level less than 0.5 i.e. there is almost zero transcription events for such low level transcriptions are rarely detectable with our Rpb1 data under 2MAD cutoff. Therefore, the skewness of peaks could be helpful in assigning Tbp1 binding to annotated features, particularly for the binding sites located in IGB (bidirectional intergenic) regions. Three examples of Tbp1 binding patterns and Rpb1 occupancies with two repeats in IGU+ (IGU between forward strand genes), IGU- (IGU between reverse strand genes) and IGB regions are shown in Figure 6.

**Figure 7:**
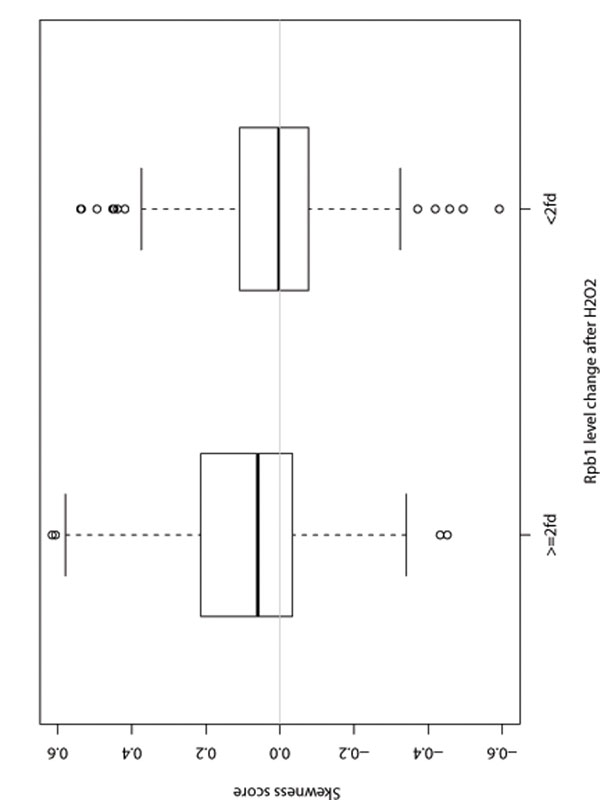
The skewness score of Atf1 and Pcr1 binding for stress response genes (more than 2 fold Rpb1 change after H_2_O_2_ treatment) and other genes (less than 2 fold Rpb1 change after H_2_O_2_ treatment). The sign of skewness score for negative strand gene bindings are changed.

**Figure 8:**
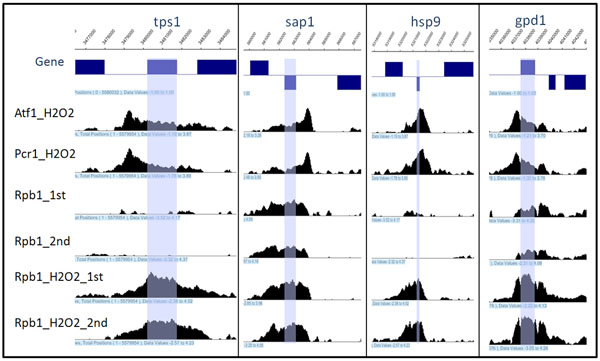
Four examples of Atf1 and Pcr1 binding patterns and Rpb1 (with and without H_2_O_2_ treatment) occupancies with two repeats. The blue boxes in first track indicate the gene ORF regions, and the vertical light blue bars indicate the stress response gene ORF regions.

Atf1 and Pcr1 bindings also display more skewed patterns at promoters of the stress response genes (more than 2 fold Rpb1 change after H_2_O_2_ treatment). As shown in Figure 7, the average skewness score of stress response gene bindings are positive (the sign of skewness score for negative strand gene bindings are changed) and the average skewness score of other genes bindings equal to 0 (*p*-value=0.007204). Four genes selected in top 20 Atf1/Pcr1-bound genes list [9] as examples are shown in Figure 8.

## Conclusions

We developed a statistical procedure to characterize binding events of DNA-interacting proteins especially transcription factors from high density ChIP-chip tiling array data. The signal regions are detected using moving window binomial analysis and the binding events are characterized by two shape parameters, kurtosis and skewness.

We applied our method to ChIP-chip data of TATA box binding protein (Tbp1), Atf1, Pcr1 and Rpb1 in *S.pombe*. We found that Tbp1 tends to have more sharp peaked occupancies than Rpb1 that indicates our methods can efficiently distinguish mostly localized DNA-protein bindings and the scattered DNA-protein interactions. We should notice even Tbp1 also has flat occupancies, that maybe due to the interaction of Tbp1 with RNA polymerase complex are maintained after the event of transcription initiation since other studies have reported there is no paused Pol II observed at the promoter regions in yeast [12]. It is also possible that Tbp1 or other components of TFIID may contribute to transcription elongation. The specific wet lab experiments may be designed to validate this assumption.

The two shape parameters of the signal regions, kurtosis and skewness, can characterize the binding patterns and the associated biology. We used kurtosis to classify the regions into peak and flat regions. The peak regions mostly fall into the promoters and the flat regions are mostly very large and cover the coding regions. We have demonstrated that the binding pattern of the peak regions in promoter regions are skewed to the downstream genes if they are transcribed, and hence the skewness can help us to predict whether the downstream gene is transcribed and to assign the binding sites to genes in the bidirectional intergenic regions.

Our method is applicable not only to ChIP-chip data, but can also be adopted to other datasets with similar goal. For example, ChIP-seq and ChIP-chip measure same signals but with different techniques. The binding patterns for ChIP-seq data should be similar to ChIP-chip data, so the peakedness and skewness assessment can be used for further analysis. Our method can be extended to other tiling array data if the patterns of the signal regions are important to the corresponding studies.

## Competing interests

The authors declare that they have no competing interests.

## Authors' contributions

RKM Karuturi and J. Li proposed the project. J. Li, L. Zhu, and RKM Karuturi developed the model, analyzed the data and wrote the paper. M. Eshaghi and J. Liu performed the ChIP-chip experiments. All the authors evaluated the results and approved the manuscript.
